# Behavioral Test Scores Could Be Linked to the Protein Expression Values of p62 and GLAST in the Brains of Mice with Neuropsychiatric Disorder-Related Behaviors

**DOI:** 10.3390/biology13121039

**Published:** 2024-12-11

**Authors:** Yuka Ikeda, Moeka Nakashima, Sayuri Yoshikawa, Kurumi Taniguchi, Naoko Suga, Satoru Matsuda

**Affiliations:** Department of Food Science and Nutrition, Nara Women’s University, Kita-Uoya Nishimachi, Nara 630-8506, Japan

**Keywords:** neuropsychiatric disorder, schizophrenia, behavioral test, poly I:C, sodium dextran sulfate, metformin, p62, GLAST

## Abstract

Neuropsychiatric disorders are a public health concern, in which diagnosis and prognosis may be based on clinical symptoms that might often diverge across individuals. Schizophrenia is a major neuropsychiatric disorder, which may affect millions worldwide. However, the biochemical alterations of this disorder have not been comprehensively distinguished. In addition, there is less confidence in finding specific biomarkers for neuropsychiatric disorders, including schizophrenia, but rather a specific characteristic behavioral pattern. In general, maternal immune activation is considered to be one of the important factors in the development of neuropsychiatric disorders. Here, a mouse model of neuropsychiatric disorders was created, in which poly I:C, sodium dextran sulfate (DSS), and κ-carrageenan (CGN) were utilized for maternal immune activation during the pregnancies of mother mice. Subsequently, we examined the link between biochemical changes in p62 and/or glutamate aspartate transporter (GLAST) in the brains of offspring mice and the alteration in their experimental behavior scores. Furthermore, a therapeutic study was conducted on these neuropsychiatric disorder model mice using butyric acid, piceid, and metformin. It was found that some molecules could effectively improve the behavioral scores of neuropsychiatric model mice. Importantly, significant correlations between certain behavioral scores and p62 protein expression, as well as between the scores and GLAST expression, were recognized. This is the first report of a significant correlation between pathological behaviors and biochemical alterations in neuropsychiatric disorder model animals. This concept could contribute to the development of innovative treatments to at least ameliorate the symptoms of several psychiatric disorders.

## Simple Summary

This study addresses the logical deliberation regarding the pathogenesis, behavioral appearances, biochemical characteristics, and reversibility of symptoms of psychiatric disorders through a dietary intervention. A mouse model of neuropsychiatric disorder was originally created with poly I:C, sodium dextran sulfate (DSS), κ-carrageenan (CGN), and Di-(2-ethylhexyl) phthalate (DEHP) usage, in which a noteworthy connection was made between alterations in p62/GLAST protein expression in mouse brains and variations in experimental behavioral test scores. In addition, a potential therapeutic study by means of dietary intervention was performed using low doses of butyrate, trehalose, and piceid. Subsequently, it was shown that these orally available molecules could effectively improve psychiatric behavior as well as the biochemical alterations in p62/GLAST in mouse brains.

## 1. Introduction

Neuropsychiatric disorders are worldwide public health concerns [1]. Schizophrenia is one of the more well-known neuropsychiatric disorders with the most severe symptoms, estimated to affect approximately 1% of the human population, which is also complicated by comorbid drug use problems [2]. Schizophrenia is regularly characterized by negative, positive, and cognitive symptoms [3]. Interestingly, schizophrenia is 1.4-times more frequently diagnosed in males than females [4]. Male patients show an earlier age of onset, decreased social functioning, and worse negative but decreased depressive symptoms compared with females [5]. However, sex differences in its cellular and molecular mechanisms remain mostly undefined. A leading theory about the etiology of schizophrenia is the dopaminergic hypothesis, which suggests that this disorder might be caused by a dopamine imbalance, which is supported by the beneficial therapeutic effects of anti-dopaminergic drugs [6]. The hypofunction hypothesis of the N-methyl-d-aspartate (NMDA) receptor may suggest a complementary elucidation of the etiology of schizophrenia [7]. Patients with schizophrenia may show a reduced expression of the NMDA receptor in the prefrontal cortex [8], which seems to also be related to the imbalance of dopamine levels [9].

It has been shown that maternal immune activation is a potential risk factor for schizophrenia [10]. Therefore, maternal immune activation using the viral RNA imitator polyriboinosinic–polyribocytidilic acid (poly I:C) during prenatal development could serve as a broadly employed animal model to create behavioral alterations reminiscent of schizophrenia [11]. These behavioral alterations reminiscent of schizophrenia may include deficits in cognition, sensorimotor gating, and acoustic startle inhibition. Later behavioral alterations in poly I:C model mice could be prevented if some interventions are performed prior to symptom manifestation [12,13]. For the disease evaluation of schizophrenia, several behavioral examinations, such as the modified forced swim test, can be regarded as appropriate tests for animal models of schizophrenia, which may be thought to imitate psychiatric symptoms [14]. Therefore, the poly I:C rodent model may be used as a neurodevelopmental paradigm of neuropsychiatric disorders based on prenatal exposure to a virus-imitating element such as poly I:C, which might lead to the development of schizophrenia-like behaviors in offspring comparable to those designated in patients [15]. In addition, several behavioral examinations related to impairments in neuropsychiatric disorders have been recorded in offspring treated with poly I:C [16,17], suggesting that patients with neuropsychiatric disorders might exhibit alterations in multisensory integration [18]. Therefore, prenatal inflammation has also been considered as a risk factor for neuropsychiatric disorders [19]. It may be of interest whether the intake of some drugs with anti-oxidative and anti-inflammatory properties could neutralize the later development of neuropsychiatric disorder-related consequences. For example, N-acetylcysteine (NAC) might be suggested as a supplement for high-risk pregnancies, as it may considerably improve neonatal consequences [20]. Taken together, the poly I:C model animal might be useful for studying the developmental factors that may alter multisensory integration in neuropsychiatric disorders [21].

At molecular levels in the brain, it has been shown that the autophagic dysregulation of p62 might potentially lead to cognitive impairment across brain conditions [22]. In addition, p62 protein expression was found to be upregulated in cultured neurons isolated from schizophrenic material [23] and in brain samples from a mouse model of schizophrenia [24]. Therefore, p62 protein levels may provide a biomarker for the potential target of therapeutic interventions against symptoms of schizophrenia [22]. Interestingly, Di-(2-ethylhexyl) phthalate (DEHP) could increase the number of autophagosomes and the levels of the autophagy marker p62, which is known to impair testicular functions and/or reproduction [25]. In addition, the DEHP has the ability to cross the blood–brain barrier, which can increase the proliferation of astrocytes in the brain [26]. Abnormalities in the regulatory components of the glutamate system might be important risk factors for schizophrenia. It has been suggested that the dysregulation of the glutamate aspartate transporter (GLAST) may also play a significant role in the neuropathogenesis of various neurological disorders, including epilepsy, autism, and schizophrenia [27]. Increased incidence of a rare genetic variant in the human gene encoding GLAST has been detected in schizophrenia patients [28]. In addition, it has been shown that *GLAST* knockout (KO) mice may display exaggerated locomotor activity in response to the administration of NMDA antagonist [29]. Furthermore, *GLAST* KO mice could exhibit phenotypic abnormalities thought to improve symptoms of schizophrenia [30].

Although the detection of predictive biomarkers has been explored for a psychiatric disorder, there is still a need for a precise clinical examination of the diagnosis of neuropsychiatric disorders including schizophrenia at an early stage. In addition, the biochemical changes in the brain have not been comprehensively characterized up to the present time. For example, there is almost no confident specific biochemical marker for neuropsychiatric disorders including schizophrenia. In the current study, therefore, we aimed to connect some neurobiochemical changes in p62 and GLAST in the poly I:C-induced model mice underlying a neuropsychiatric disorder toward the alteration of several behavioral scores with neuropsychiatric disorders. From the results of many preliminary experiments with various behavioral examinations and/or diverse possible scorings, we selected and utilized the presented original behavioral examinations and Western blot analysis of p62 and GLAST proteins, in order to investigate the probable levels of an applicable relationship.

## 2. Materials and Methods

### 2.1. Mice

**Figure 1:**
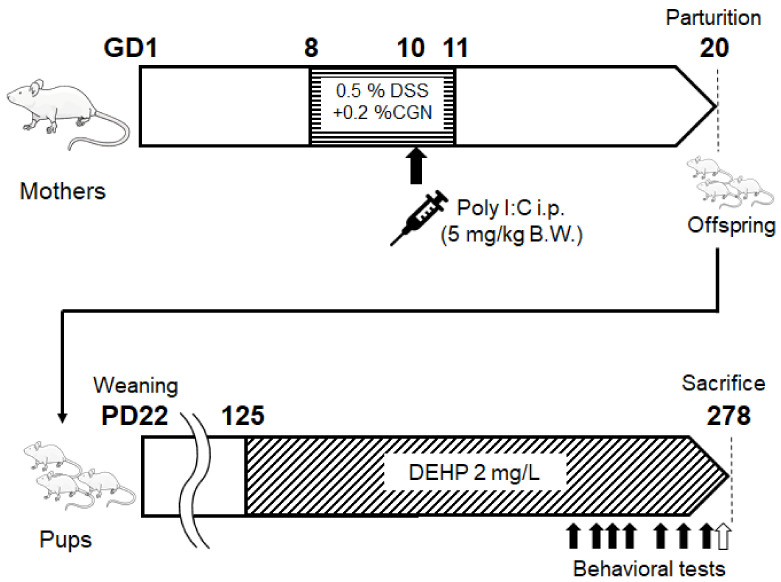
Preparation of psychiatric disorders model mice with behavioral alteration: Schematic representation of the treatment design for making schizophrenia like disorder model mice is shown. Briefly, female ICR mice were mated with male ICR mice. After the day when the vaginal plug was confirmed as her pregnancy (GD1), the mother mice received with DSS + CGN water from GD 8 to 11 days. And 5 mg/kg body weight of Poly I:C was administered intraperitoneally at the GD10. Several numbers of pups were born at PD1. The pup mice were separated from their mothers at PD22. Pup mice were given 2 mg/L DEHP from PD125 at least to PD215. After that, mice were conducted for eight times of behavioral test. Black arrows show the day of behavioral tests. DSS: Dextran sodium sulfate, CGN: κ-Carrageenan.

Male and female mice established in Institute for Cancer Research (ICR mice) (4 weeks old) were purchased from Charles River Laboratories Japan, Inc. (Kanagawa, Japan). At the age of 6 weeks after acclimation, female ICR mice were mated with male ICR mice, and the day when the vaginal plug was confirmed was defined as the first day of pregnancy (GD1). The mother mice received DSS + CGN water, which was dissolved in 0.5% (*w*/*v*) sodium dextran sulfate 5000 (DSS) and 0.2% (*w*/*v*) κ-Carrageenan (CGN) in GD 8 to 11. And 5 mg/kg B.W. Poly I:C was administered intraperitoneally on GD10. The day mice were born was defined as PD1, and on day 22 (PD22), mice were separated from their mothers and grouped in net cages separately for males and females. From PD 125, mice were given 2 mg/L DEHP, which was freely available for mice. There was no other water supply. Then, three types of behavioral tests, a descent step test, a modified three-chamber test, and a light/dark room test, were conducted eight times. After the behavioral tests, some of the mice were dissected, and we collected liver, kidney, and brain. For the therapeutic study, twenty-three male mice (SZ), shown in Figure 1, were utilized for further experiments.

The mice were housed in an environmentally controlled room, at approximately 20 °C and 60% humidity with a 12 h light/dark cycle (lights on at 07:00 and off at 19:00). The care and treatment of the experimental animals conformed with the guidelines for the ethical treatment of laboratory animals established by Nara Women’s University (Nara, Japan) (Approval No. 19-02).

### 2.2. Materials

Poly I:C, DEHP, and dextran sulfate sodium (DSS, MW5000) were purchased from Fujifilm Wako Pure Chemical Corporation (Osaka, Japan). κ-Carrageenan (CGN) was obtained from Tokyo Chemical Industry Co., Ltd. (Tokyo, Japan). Poly I:C was dissolved in saline. DEHP, DSS, and CGN were diluted with sterile water for mice to drink. Butyric acid, metformin, trehalose, and piceid were purchased from Fujifilm Wako Pure Chemicals Corporation (Osaka, Japan). They were also dissolved in sterile water for the mice to drink. The other reagents were obtained from FUJIFILM Wako Pure Chemical Co. (Osaka, Japan).

### 2.3. Behavioral Tests

The score of behavioral tests is shown in Table 1.

#### 2.3.1. Descent Step Test

**Figure 2:**
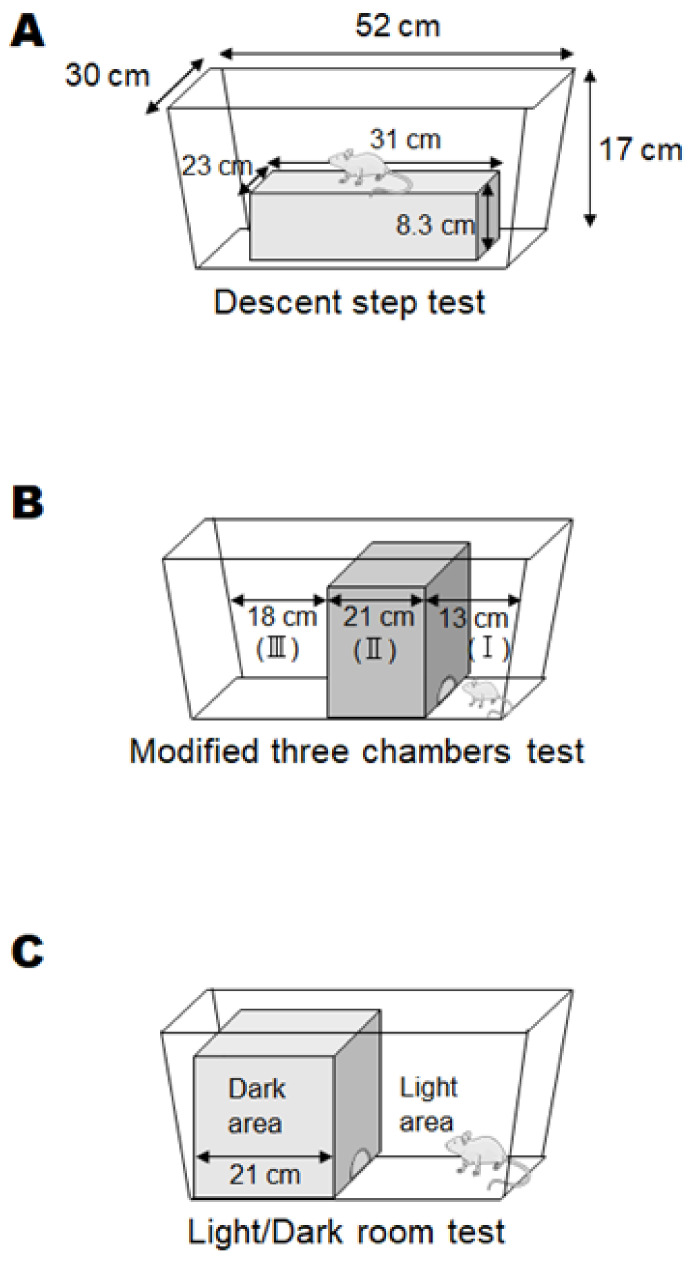
Behavioral tests: (**A**) The image of the descent step test. The mouse placed on the box and be measured whether or not the mice descended from the box within one minute. (**B**) The image of the modified three chambers test. At first, the mouse was placed in (I), and we measured which chamber the mouse was inside after 25 s. (**C**) The image of the light/dark room test. Mice were allowed to explore freely for 2 min, and we measured the time spent in the light area.

A box (23 cm × 31 cm × 8.3 cm) was placed in the center of a plastic case (30 cm × 52 cm × 17 cm), and each mouse was placed on the box. After that, we measured whether or not the mouse descended from the box within one minute (Figure 2A).

#### 2.3.2. Modified Three-Chambers Test

The field of the plastic case was divided into three compartments with boxes, and the central compartment was dark (27.5 cm × 21 cm). At first, each mouse was placed in a narrow bright place (30 cm × 13 cm), and chambers were set as (I), (II), and (III) in order of proximity. The size of the dark entrance was 4 cm × 2.5 cm. The score was determined by the position mice were in 25 s after the start (Figure 2B).

#### 2.3.3. Light/Dark Room Test

A dark space (27.5 cm × 21 cm) and a light space (30 cm × 31 cm) were set up in a box in a field of a plastic case, and the boundary between the light space and the dark space was used as the starting point. The size of the dark entrance was 4 cm × 2.5 cm. Mice were allowed to explore freely for 2 min, and the time spent in the light was measured (Figure 2C).

### 2.4. Western Blotting

To extract protein, the whole brain was homogenized with RIPA buffer. The homogenates were centrifuged to obtain supernatants (Tabletop micro-cooled centrifuge Model3500, Kubota, Tokyo, Japan). The supernatants were mixed with sample buffer and adjusted 1 mg/mL protein concentration. We used SDS-PAGE to separate proteins and transfer them to membranes (Immobilon-P, Merck KGaA, Darmstadt, Germany). These were blocked with 3% skim milk and then reacted with primary antibodies SQSTM1/p62 polyclonal antibody (Cosmobio) or GLAST polyclonal antibody (Cosmobio) at 1 h and peroxidase-conjugated goat anti-rabbit secondary antibodies (Cell Signaling) at 1 h. Proteins were detected by ImageQuant LAS500 (GE Healthcare Japan Com., Tokyo, Japan). Each detected band was quantified by ImageJ, and the relative ratio of protein expression was analyzed using GAPDH (Glyceraldehyde 3-phosphate dehydrogenase, FUJIFILM Wako Pure Chemicals Co., Osaka, Japan) as an internal control protein. The intensities of the detected bands were calculated using ImageJ (v.1) software.

### 2.5. Statistical Analyses

Animal data are expressed as the mean ± standard error (SE). All data were analyzed by Pearson’s correlation analysis. *p* < 0.05 was considered a statistically significant difference. All statistical analyses were performed using GraphPad Prism version 5.0 (GraphPad Software, Inc., San Diego, CA, USA). As for the analysis of the correlation between PBI score and p62 protein expression, alteration in the PBI score (score change) was calculated by the following equation: score change = (average of three times of PBI score before the treatment) − (average of at least five times of PBI score after the treatment).

## 3. Results

**Figure 3:**
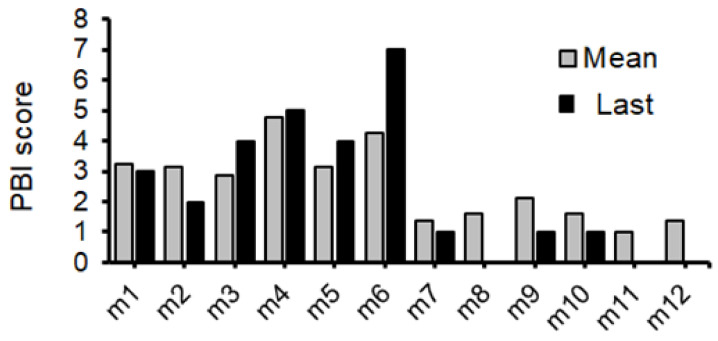
The psychological behavior index (PBI) scores of each mouse: The PBI score was calculated with a sum of three behavioral tests scores for each of individual pups. The grey bar shows the mean value of 8 times of PBI scores during the whole preliminary study. Black bar shows the last behavioral test score at PD278 before dissection of mice. In consequence of the death of pups before final dissection, some black bars are missing (at m8, m11, and m12).

**Figure 4:**
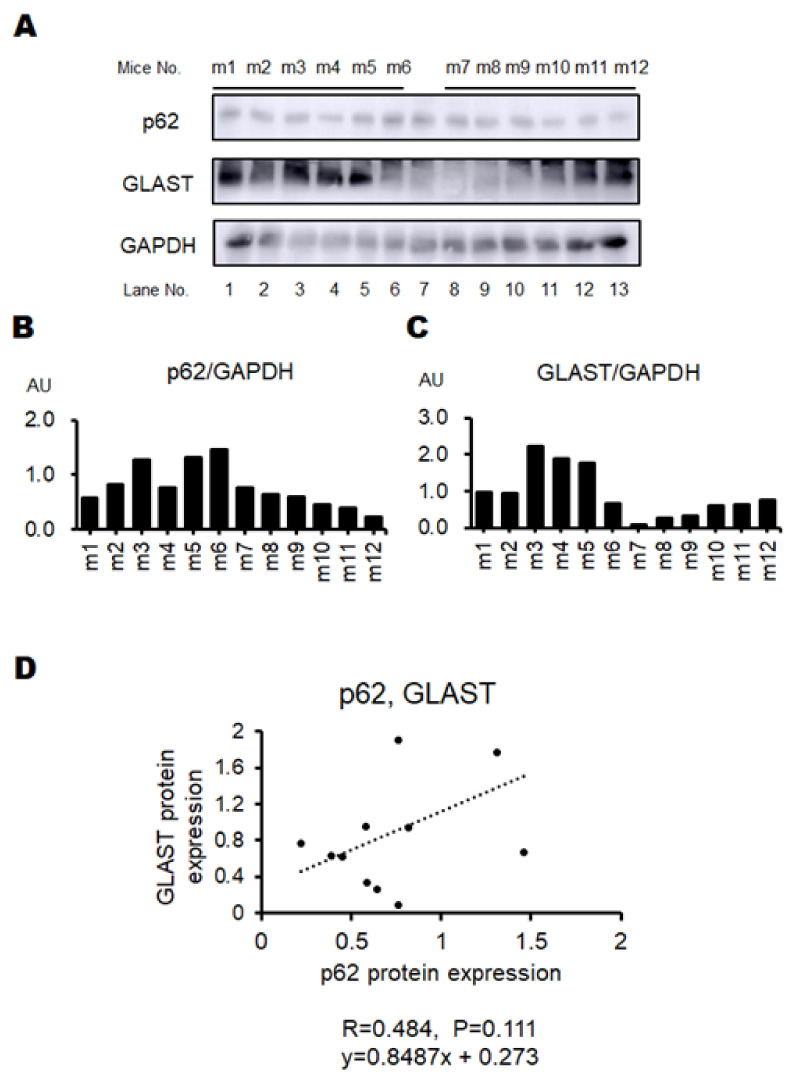
Some protein expressions in the brain: (**A**) The representative image of p62, GLAST, and GAPDH expression in the brain of individual mouse. The lane 7 shows the result of untreated standard mouse (**B**) The protein expression of p62 was measured and normalized to GAPDH by Western blot. (**C**) The protein expression of GLAST was measured and normalized to GAPDH by Western blot. GLAST; a glutamate transporter protein. (**D**) Positive correlation between the p62 and GLAST protein expression. r = 0.484, *p* = 0.111, y = 0.8487x + 0.273.

**Figure 5:**
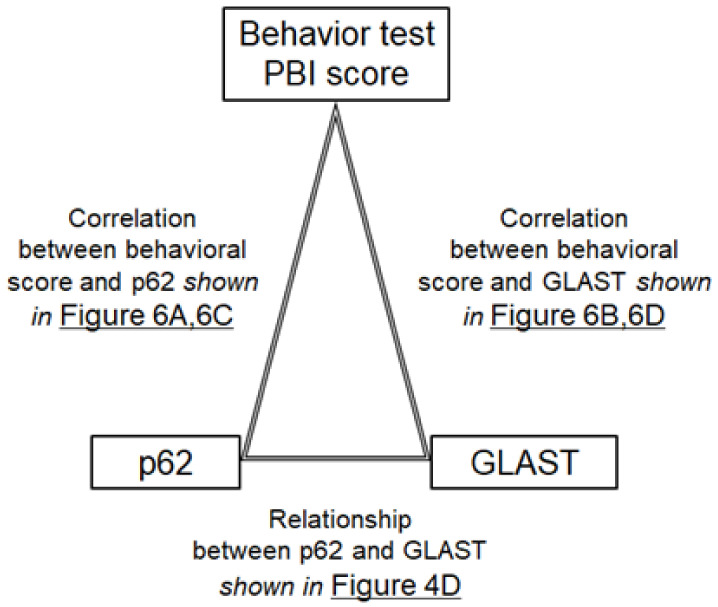
Overview of correlation analyses for the preset preliminary study: The image of the relation of behavioral tests, p62, and GLAST. The correlation of behavioral tests and the p62 expression is shown in Figure 6B,D. The correlation of behavioral tests and the GLAST expression is shown in Figure 6A,C. The correlation between p62 and GLAST expression is shown in Figure 4D.

**Figure 6:**
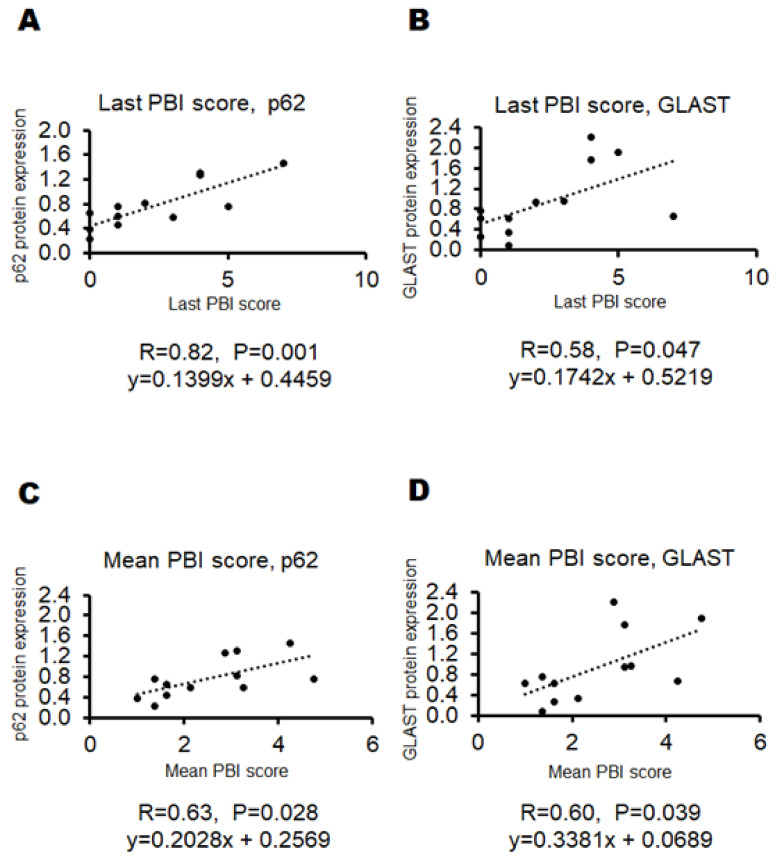
The correlation between behavioral tests score and the protein expression of p62 and GLAST: (**A**) Positive correlation between the last behavioral test score and p62 protein expression. r = 0.82, *p* = 0.001, y = 0.1399x + 0.4459 (**B**) Positive correlation between the last behavioral test score and GLAST protein expression. r = 0.58, *p* = 0.047, y = 0.1742x + 0.5219 (**C**) Positive correlation between the mean behavioral test score and p62 protein expression. r = 0.63, *p* = 0.028, y = 0.2028x + 0.2569 (**D**) Positive correlation between the mean behavioral test score and GLAST protein expression. r = 0.60, *p* = 0.039, y = 0.3381x + 0.0689.

Certain behavioral test scores showing an accurate pathological condition without any burden to patients might encourage the development of a good treatment against neuropsychiatric disorders that would significantly exhibit some biochemical alteration in the brain. For this purpose, we struggled to look for a specific behavioral test which could represent the objective status of neuropsychiatric disorder levels. Here, a result of the preliminary experiment with a certain behavioral test has been demonstrated for the comprehension of the relationship between the test score and the biochemical protein expression levels related to the pathology of the disease. To facilitate the development of neuropathological disorders in offspring, some modifications have been employed in addition to the basic poly I:C method for making model mice with schizophrenia-like behaviors. First, per oral administration of low-molecular-weight sodium dextran sulfate (DSS), κ-Carrageenan (CGN) was added to the pregnant maternal mice for enhancing the inflammation levels [31]. In addition, puppies were forced to drink DEHP water, which might efficiently cause neurobehavioral disorders in offspring with the increased level of oxidative stress [32]. Remarkably, this is a new mouse model that has not been used previously. However, note that it was impossible to make all the mice treated have psychiatric behavior. Three behavioral tests were originally designed for the present study with several modifications from the literature [15,24,33] (Figure 2, Table 1). The psychological behavior index (PBI) score was calculated with a sum of three behavioral tests scores for each of individual pups (Figure 3, Table 1). Behavioral tests were conducted eight times during the whole experiment period for an individual (Supplementary Data, S1 and S2). The average scores and the last score during the period are shown (Figure 3). Note that zero or low values of the PBI score may be found in untreated and/or standard offspring (*n* > 10) (personal communication). After documented PBI scores, all mice were sacrificed and examined for protein expressions on their whole brain. The protein expression levels of p62, GLAST, and glyceraldehyde 3-phosphate dehydrogenase (GAPDH) in each mouse brain are shown in Figure 4A–C. There was no significant relationship between p62 and GLAST protein expressions (*p*: 0.111) (Figure 4D). Interestingly, the correlation between the last PBI score and p62 protein expression (*p*: 0.001) as well as the correlation between last PBI score and GLAST protein expression (*p*: 0.047) were significantly identified (Figure 5 and Figure 6A–D). The results presented here can be considered as preliminary data, however, which may suggest that a behavioral test score might be associated with the protein expression levels of p62 and/or GLAST in the brain of mice with probable neuropsychiatric disorders.

**Figure 7:**
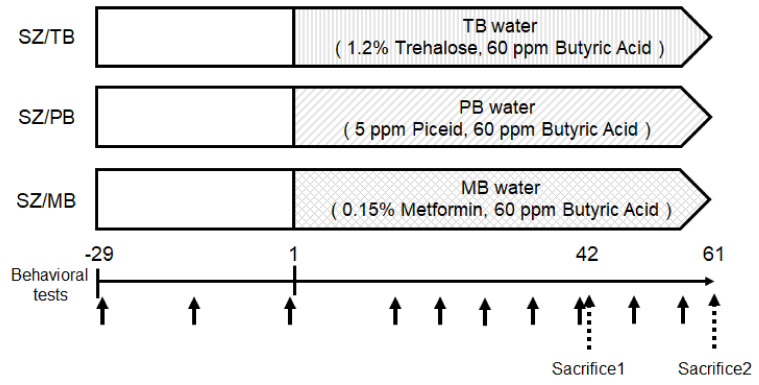
Study design. Twenty-three psychiatric disorder model mice (SZ) previously made as Figure 1 were divided into three groups of SZ/TB (1.2% Trehalose, 60 ppm Butyric Acid), SZ/PB (5 ppm Piceid, 60 ppm Butyric Acid), SZ/MB (0.15% Metformin, 60 ppm Butyric Acid) and sacrificed on day 42 and 61. All mice were conducted for behavioral tests on the arrowhead days. The day of behavioral tests is shown a black arrow. Dotted arrows show the day of sacrifice.

**Figure 8:**
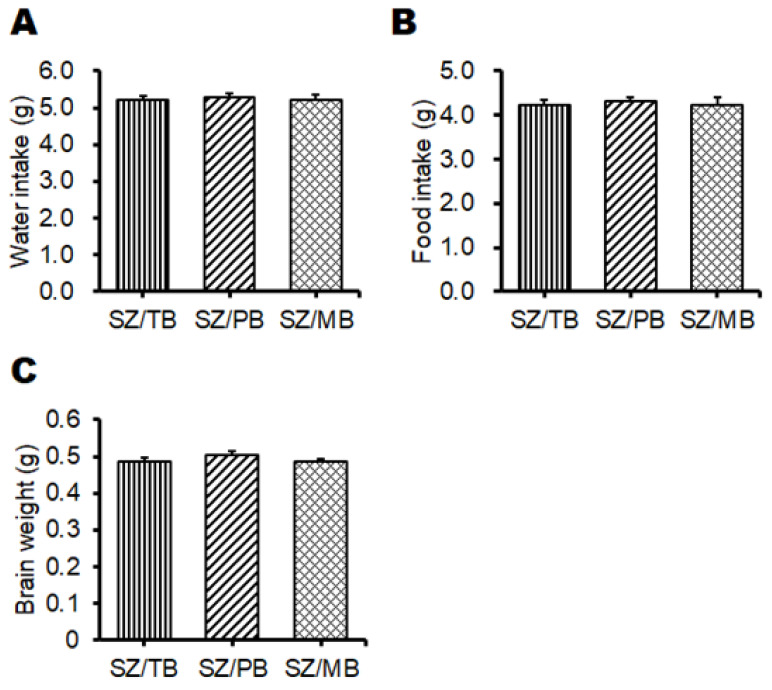
Water intake, food intake, and the brain weight in mice: (**A**) Water intake was quantified once a week throughout the experiment. SZ/TB group (gray), SZ/PB group (right-upper diagonal), SZ/MB group (mesh pattern). Values are expressed as the mean ± SE, n = 7/group. The data were tested by one-way ANOVA. (**B**) Food intake was quantified once a week throughout the experiment. SZ/TB group (gray), SZ/PB group (right-upper diagonal), SZ/MB group (mesh pattern). Values are expressed as the mean ± SE, n = 7/group. The data were tested by one-way ANOVA. (**C**) The brain weight was quantified after the sacrifice of the mouse. SZ/TB group (gray), SZ/PB group (right-upper diagonal), SZ/MB group (mesh pattern). Values are expressed as the mean ± SE. The data were tested by one-way ANOVA.

**Figure 9:**
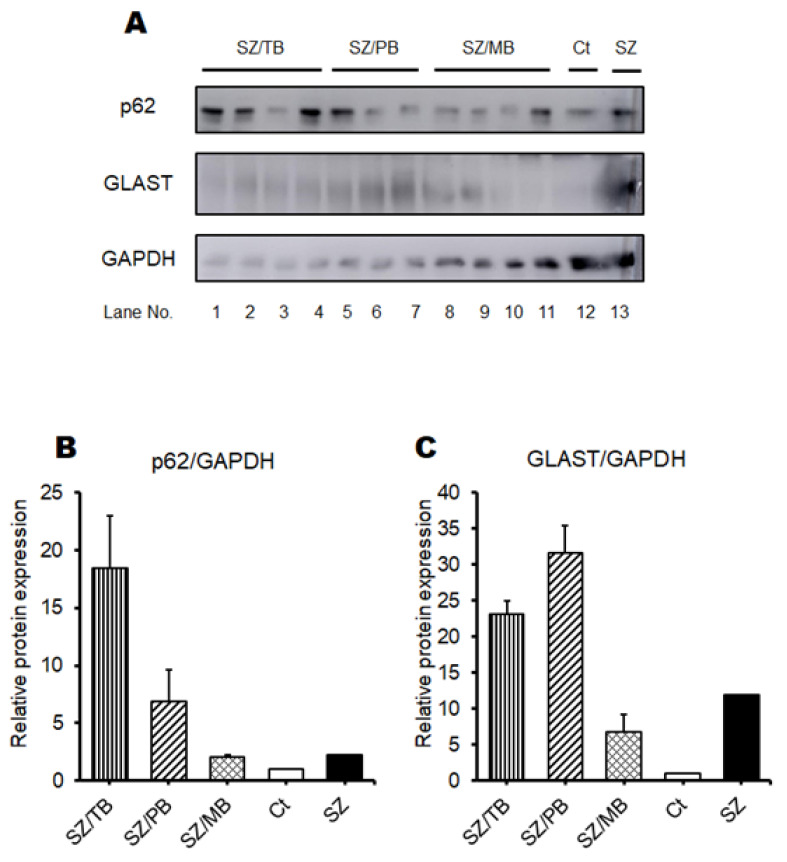
The expression of p62 and GLAST proteins in the brain: (**A**) The image of p62, GLAST and GAPDH expression by Western blot analysis. Ct shows the untreated normal mouse, whereas SZ shows psychiatric disorder model mice previously made as shown in Figure 1. (**B**) The protein expression of p62 (60 kDa) was quantified and normalized to that of GAPDH by Western blot. SZ/TB group (gray), SZ/PB group (right-upper diagonal), SZ/MB group (mesh pattern), Ct (white), SZ (black). Values are expressed as the mean ± SE. The data were tested by one-way ANOVA. (**C**) The protein expression of GALST (98 kDa) was quantified and normalized to that of GAPDH by Western blot. SZ/TB group (gray), SZ/PB group (right-upper diagonal), SZ/MB group (mesh pattern), Ct (white), SZ (black). Values are expressed as the mean ± SE. The data were tested by one-way ANOVA.

**Figure 10:**
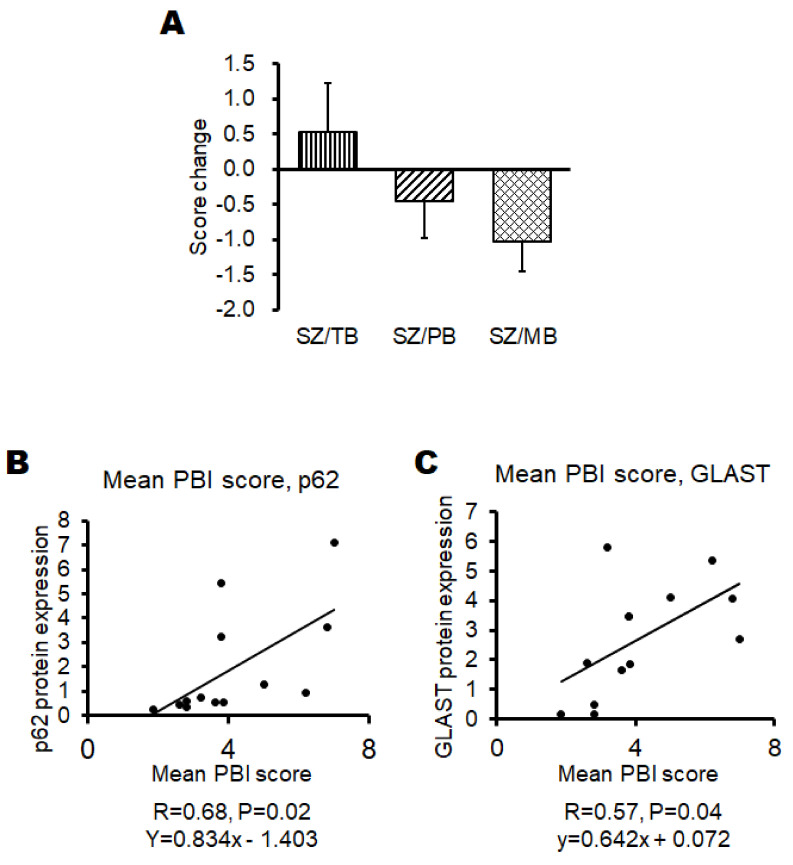
Improvement in the PBI score and the correlation between test score and the protein expression of p62/GLAST: (**A**) Alteration in the PBI score (score change) was calculated by the following equation, score change = (average of three times of PBI score before the treatment) − (average of at least five times of PBI score after the treatment). Value of the score change is expressed as the mean ± SE. SZ/TB group (gray), SZ/PB group (right-upper diagonal), SZ/MB group (mesh pattern), Ct (white), SZ (black). (**B**) Positive correlation between the mean behavioral test score and p62 protein expression. r = 0.68, *p* = 0.02, y = 0.834x − 1.403. (**C**) Positive correlation between the mean behavioral test score and GLAST protein expression. r = 0.57, *p* = 0.04, y = 0.642x + 0.072.

Next, we further examined the relationship between the behavioral test score and the p62/GLAST protein expression levels in therapy experiments against these possible neuropsychiatric disorder mice. It has been reported that autophagy-related materials, such as metformin, butyrate, trehalose, and piceid, may be beneficial for the treatment of schizophrenia [34,35,36]. Therefore, we employed our model mice originally made for a neuropsychiatric disorder whether these molecules could contribute to an improvement in the neuropsychiatric behavioral symptoms of mice. In the preliminarily conducted experiments, behavioral tests indicated that metformin or butyrate could improve the behavioral score of model mice to some extent. Consistently, it has been shown that schizophrenia may be characterized by the depletion of anti-inflammatory butyrate-producing genera [37]. Expecting additional favorable effects, therefore, we used three concomitant combinations of butyrate and other molecules for the treatment of model mice. An overview of this study design is shown in Figure 7. In the present experimental condition, there was no significant difference in water intake (Figure 8A), food intake (Figure 8B), body weight (unpublished data, and personal communication), and brain weight (Figure 8C) alterations among groups. In addition, no mice died during the experiment. The p62 and GLAST protein expression of the SZ groups was higher than those of the Ct group, but the SZ/MB group showed similar levels of p62 protein expression to the Ct group (Figure 9A–C). Additionally, the lowest protein expression of p62 and GLAST in the three combination treatment groups was exhibited by the SZ/MB group (Figure 9A–C). The efficacy outcome was assessed in terms of the PBI score reduction. The most efficient combination was also exhibited by the SZ/MB group (Figure 10A). Importantly, the correlation between PBI score and p62 protein expression (*p*: 0.02) as well as the correlation between PBI score and GLAST protein expression (*p*: 0.04) were significantly identified again (Figure 10A,B).

## 4. Discussion

One of the goals in this study was effectively to produce a mouse model of neuropsychiatric disorders after exposure to maternal immune activation via the viral nucleic acid imitators, including the poly I:C. Almost consistent with earlier reports, we observed that acute poly I:C administered at PD10 plus DSS/CGN supply could elicit significant alterations in behavioral test scores in our original tests for adult offspring animals. The behavioral experiments also revealed an array of long-term changes in the offspring following poly I:C, DSS/CGN, and DEHP treatments. In line with this, some studies have proved object recognition deficits in mice prenatally exposed to the similar poly I:C and lipopolysaccharide (LPS) treatment [38,39]. The effects of these poly I:C treatments on the alteration in behaviors observed in the present study had a tendency to increase in male mice, whereas female mice showed a reduction in effects (personal communication). Interestingly, sex differences in the effects of prenatal infection for cognition have also been described for fear conditioning in rodents [40,41]. In addition, previous reports suggest that the phase of the estrous cycle of female rodent animals may affect the object recognition during upper levels of estrogen and/or progesterone of female animals [42].

In neurons derived from schizophrenia patients, the sensitivity to PI3K/AKT/GSK3 signaling might be changed [43], which may be involved in the pathogenesis of schizophrenia [44]. In addition, the AKT activity has been shown to decrease in certain brain regions of patients with major depressive disorder and/or schizophrenia [45]. Interestingly, anti-inflammatory pregnenolone has been indicated to control schizophrenia-like behaviors via the modulation of AKT signaling [46]. It has been shown that the PI3K/AKT signaling pathway might be involved in autophagy [47]. Therefore, autophagy might also be involved in the development of schizophrenia [48]. Autophagy is a membrane trafficking machinery responsible for degrading damaged proteins, lipids, and/or organelles in the lysosome [49]. Remarkably, neuronal autophagy may be related to the cognitive processes via the regulation of synaptic components [23,50]. As for the therapeutic study presented here, some effective molecules for the improvement of psychiatric behavior were piceid, metformin, and butyrate (Figure 10). These molecules have been reported, conceivably, to be involved in the modulation of autophagy [34,35,36]. In line with this, the intracellular autophagic activity could control the p62 protein levels [51]. It has been proposed that elevated p62 levels may have functional consequences on the neurotransmission, which might explain the behavioral changes relevant to schizophrenia [23,52]. Fortunately, it has been shown that the protein expression levels of p62 seem to be significantly correlated with the score of our behavioral tests. Aberrations in regulatory components of the glutamatergic system could also be important risk factors for schizophrenia, which might be regulated by a family of glutamate transporters, including GLAST or excitatory amino-acid transporter 1. The incidence of a genetic variant may be increased in the human gene encoding GLAST within schizophrenia patients [28,53]. Interestingly, some genetic variants may impair metabolic functions of astrocytes and might lead to cognitive dysfunction [54]. In addition, it has been revealed that the GLAST knockout (KO) mice could exhibit exaggerated locomotor activity [28,55], which may be a model for positive symptoms of schizophrenia. Therefore, the roles of GLAST protein might be involved in the certain behavior relevant to the symptoms of schizophrenia [30,53,56]. Fortunately, again, the expression levels of GLAST protein seem to also be correlated with the scores of our behavioral tests. These data provide the first demonstration of the correlations between behavioral scores and the biochemical alterations in p62 and/or GLAST expression as a result of a prenatal immune challenge, which may support the hypothesis that prenatal infection disrupts processing within the prefrontal cortex, consistent with findings regarding the pathophysiology of neuropsychiatric disorders, including schizophrenia and/or autism [56,57].

Again, the present experiments demonstrate that a prenatal immune challenge results in behavioral alterations in mice similar to those reported in patients with neurodevelopmental psychiatric disorders such as schizophrenia. Additionally, the most common pharmacological strategy to elicit schizophrenia-like behaviors might be based on the inhibition of the N-methyl-D-aspartate (NMDA) receptor [58]. A non-competitive NMDA receptor antagonist could mimic the hypofunction of the NMDA receptor, which might lead to behavioral effects reminiscent of schizophrenia [59]. The predictive rationality of that model is well known due to the effective administration of atypical antipsychotic drugs. No other drugs such as antidepressants could invert the behavioral changes in that model. Whatever the neuropsychiatric disorder is, the approach presented here would make it easy and/or cost effective to determine whether some preventive materials, including the use of antipsychotics, hormonal agents, and/or anti-inflammatory drugs, should be applied to the adolescent poly I:C animals presented here [60,61]. Continuous and live evaluations might be possible for the evaluation of therapeutic intervention to the model animal of neuropsychiatric disorders. There are new methods for this.

The clinical symptoms of neuropathological disorders might be intricate. Our results are limited to an individual model animal, in which the phenotype may be partially present in the poly I:C-treated mice offspring. Even though the poly I:C model was well validated, there are currently no animal models which completely mimic a human neuropathological disorder. Therefore, the validity of the behavioral score should be re-evaluated with the model mice with the treatment of NMDA receptor inhibitors. Response to the administration of atypical antipsychotic drugs might also be mandatory. The communication between neurons and glial cells is also important for the appropriate function in the brain. Therefore, a histopathological study might be helpful for the elucidation of roles with the communication in distinct brain areas. In addition, a larger experimental sample size might lead to more significant outcomes. As the results of this study were attained by using a mouse model, the generalization of our findings to humans should be cautiously assessed. Studies on the mechanism of action related to the prevention of schizophrenia-like outcomes in the offspring are also necessary. In this regard, we presume that neuropsychiatric disorders might be categorized in immune-related diseases [62], whose pathogenesis may be based on the engram memory systems [63,64,65]. Forthcoming studies using a larger number of animals to address the above concerns and/or concepts would be informative. Further research will be required to identify the differences and commonalities between the neuropathological mechanisms underlying the behavioral outcomes of prenatal immune activation.

## 5. Conclusions

Taken together, a mouse model of neuropsychiatric disorders was constructed, in which poly-I:C, DSS, and CGN were used for maternal individual immune activation during the pregnancy. In addition, significant correlations between the behavioral test scores and the protein expression levels of p62 and GLAST in the whole brain of offspring mice were detected. For the evaluation of neuropsychiatric disorder-like behaviors, three behavioral tests were created. In conclusion, autophagy regulation might be involved in the occurrence and/or the development of these neuropsychiatric disorders. It was found that some combination treatment using butyric acid, piceid, and metformin could effectively improve the behavioral scores of neuropsychiatric model mice. These findings suggest that constructive behavioral tests could be effective for determining some of the brain neuropathological disorder. However, further investigation is required to fully comprehend the molecular mechanisms involved.

## Figures and Tables

**Table 1:** Behavioral test scores.

Behavioral Test		Score
Descent step test	Non-descent	0
	descent	1
Modified three chambers test	In (III) room	1
	In (I) room	2
	In (II) room	4
Light/Dark room test	Time in light area ≧60 (sec)	0
	Time in light area 40–59 (sec)	1
	Time in light area 20–39 (sec)	2
	Time in light area 0–19 (sec)	3

## Data Availability

Data are contained within the article and Supplementary Materials.

## Supplementary Materials

The following supporting information can be downloaded at: https://www.mdpi.com/article/10.3390/biology13121039/s1, S1: Results of behavioral tests; S2: Behavioral test scores.

## Abbreviations

CGN	carrageenan
CNS	central nervous system
DEHP	2-ethylhexyl phthalate
DSS	sodium dextran sulfate
FMT	fecal microbiota transplantation
GAPDH	glyceraldehyde 3-phosphate dehydrogenase
GLAST	glutamate aspartate transporter
KO	knockout
LPS	lipopolysaccharide
mRNA	messenger RNA
NAC	N-acetylcysteine
NMDA	N-methyl-d-aspartate
PBI	psychological behavior index
poly I:C	polyriboinosinic-polyribocytidilic acid
QOL	quality of life
