# Supplementary material for: Behavioral Test Scores Could Be Linked to the Protein Expression Values of p62 and GLAST in the Brains of Mice with Neuropsychiatric Disorder-Related Behaviors
Source: Biology (Basel). 2024 Dec 11;13(12):1039. doi: 10.3390/biology13121039 (PMC11672909; doi:10.3390/biology13121039)
Supplement: Supplementary file 1 [file biology-13-01039-s001.zip › biology-3224594-supplementary.pdf]

S1 Results of behavioral tests

Day		-28			-14			-1			14			21			28			35			42			48			56		
Behavioral tests		[1]	[2]	[3]	[1]	[2]	[3]	[1]	[2]	[3]	[1]	[2]	[3]	[1]	[2]	[3]	[1]	[2]	[3]	[1]	[2]	[3]	[1]	[2]	[3]	[1]	[2]	[3]	[1]	[2]	[3]
SZ/TB	m1	5	3	48	3	3	37	2	2	42	2	2	6	3	2	2	2	2	17	2	2	3	3	2	120	2	2	19	1	2	120
	m2	4	2	75	7	1	98	6	2	84	4	1	110	3	2	6	3	1	35	3	2	28	2	2	2	1	2	4	1	2	2
	m3	51	2	18	30	2	10	38	2	1	20	2	2	8	2	1	6	2	1	1	2	2	3	2	1						
	m4	×	2	120	×	1	84	×	2	73	×	1	120	×	1	81	×	1	89	17	1	102	21	1	77	6	3	50	4	1	103
	m5	×	1	24	×	3	8	×	1	8	37	2	20	6	2	1	6	2	7	4	2	9	5	2	3						
	m6	×	2	61	×	2	28	×	3	47	×	2	37	×	2	32	24	2	76	4	3	64	6	2	61						
	m7	22	2	71	25	2	48	6	2	105	18	2	105	15	2	120	4	2	85	2	3	77	2	1	69						
SZ/PB	m1	7	2	15	4	3	12	3	2	95	7	2	81	3	1	0	1	1	33	1	3	5	0	2	1	1	2	22	1	2	1
	m2	×	1	68	×	1	44	×	1	53	21	3	56	4	2	13	3	2	31	5	3	77	5	3	65	3	3	83	1	2	118
	m3	4	3	17	6	1	32	4	2	42	11	2	57	3	2	52	4	1	94	4	2	58	2	3	71						
	m4	4	1	119	34	3	57	29	1	64	9	3	85	6	2	63	3	1	47	2	1	56	6	3	75	3	3	76	2	2	56
	m5	5	2	12	6	2	4	3	2	22	5	2	11	2	2	0	2	3	1	4	2	7	2	2	9						
	m6	24	1	77	12	1	33	7	2	70	×	3	39	30	2	13	11	2	23	4	2	56	5	2	56	4	2	52	2	1	13
	m7	3	1	30	×	2	5	×	2	11	×	2	18	18	3	40	16	3	79	15	3	86	3	2	63						
SZ/MB	m1	38	2	2	22	1	11	×	1	16	5	2	18	5	2	26	3	1	77	4	3	69	3	3	67						
	m2	24	2	33	25	2	26	5	2	42	10	2	83	7	3	23	3	3	43	3	2	11	2	2	10	2	2	53	4	1	23
	m3	×	3	74	×	1	96	×	2	54	×	1	83	×	3	50	44	1	100	9	3	61	7	1	40						
	m4	12	1	94	4	2	64	3	2	7	9	1	100	6	3	41	3	1	68	6	1	59	2	1	35						
	m5	×	3	76	50	2	87	24	2	81	×	1	120	42	2	62	6	3	89	6	1	71	3	3	62						
	m6	9	2	42	37	1	44	17	1	43	17	2	32	10	2	61	8	2	45	5	2	47	2	1	43	1	1	51	1	2	68
	m7	2	2	110	3	3	84	2	3	38	3	1	69	10	2	82	4	1	74	3	1	68	1	3	68	1	1	65	3	1	55
		seconds	room	seconds																											

Behavioral Tests

[1]Descent step test : Numbers indicate seconds descent, × indicates non-descent.

[2]Modified three chambers test : Numbers indicate the room which position mice were in 25 seconds after the start.

[3]Light/Dark room test : Numbers indicate the seconds that mice spent in the light area.

S2 Behavioral test scores

SZ/PB	m1	0	4	3	0	0	3	0	4	0	0	4	0	0	2	3	0	2	2	0	0	3	0	4	3	0	4	2	0	4	3
	m2	1	2	0	1	2	1	1	2	1	0	0	1	0	4	3	0	4	2	0	0	0	0	0	0	0	0	0	0	4	0
	m3	0	0	3	0	2	2	0	4	1	0	4	1	0	4	1	0	2	0	0	4	1	0	0	0						
	m4	0	2	0	0	0	1	0	2	0	0	0	0	0	4	0	0	2	1	0	2	1	0	0	0	0	0	0	0	4	1
	m5	0	4	3	0	4	3	0	4	2	0	4	3	0	4	3	0	0	3	0	4	3	0	4	3						
	m6	0	2	0	0	2	2	0	4	0	1	0	2	0	4	3	0	4	2	0	4	1	0	4	1	0	4	1	0	2	3
	m7	0	2	2	1	4	3	1	4	3	1	4	3	0	0	1	0	0	0	0	0	0	0	4	0						
SZ/MB	m1	0	4	3	0	2	3	1	2	3	0	4	3	0	4	2	0	2	0	0	0	0	0	0	0						
	m2	0	4	2	0	4	2	0	4	1	0	4	0	0	0	2	0	0	1	0	4	3	0	4	3	0	4	1	0	2	2
	m3	1	0	0	1	2	0	1	4	1	1	2	0	1	0	1	0	2	0	0	0	0	0	2	1						
	m4	0	2	0	0	4	0	0	4	3	0	2	0	0	0	1	0	2	0	0	2	1	0	2	2						
	m5	1	0	0	0	4	0	0	4	0	1	2	0	0	4	0	0	0	0	0	2	0	0	0	0						
	m6	0	4	1	0	2	1	0	2	1	0	4	2	0	4	0	0	4	1	0	4	1	0	2	1	0	2	1	0	4	0
	m7	0	4	0	0	0	0	0	0	2	0	2	0	0	4	0	0	2	0	0	2	0	0	0	0	0	2	0	0	2	1

Score

Behavioral test	Division		Score
[1]Descent step test	Descent		0
	Non-descent		1
[2]Modified three chambers test	In (Ⅲ) room		0
	In (Ⅰ) room		2
	In (Ⅱ) room		4
[3]Light/Dark room test	Time in light area	≧60(sec)	0
		40-59(sec)	1
		20-39(sec)	2
		0-19(sec)	3
